# Editorial: Bioinformatics analysis tools for nanopore sequencing and applications

**DOI:** 10.3389/fbioe.2023.1189769

**Published:** 2023-03-28

**Authors:** Jidong Lang, Jialiang Yang, Miao Xu

**Affiliations:** ^1^ Department of Bioinformatics, Qitan Technology Co., Ltd., Beijing, China; ^2^ Geneis Beijing Co., Ltd., Beijing, China; ^3^ Broad Institute of Harvard and Massachusetts Institute of Technology, Cambridge, MA, United States

**Keywords:** nanopore sequencing technology, application analysis tools, bioinformatics, statistical analysis, deep learning methods

Nanopore sequencing technology (NST), also known as single molecule real-time sequencing technology, allows deciphering single DNA and RNA molecules without polymerase chain reaction (PCR). Currently, NST has undergone rapid development in scientific research and clinical practice, with substantial improvements made in the read length and sequencing throughput. These breakthroughs have required extensive development of experiments and bioinformatics methods to take full advantage of nanopore long-read sequencing in areas such as genomics, transcriptomics, epigenomics and epitranscriptomics (Amarasinghe et al., 2020; Wang et al., 2021). NST is currently being applied to genome assembly, full-length transcript sequencing and base-modification detection, and other specialized fields, such as rapid clinical diagnosis of pathogenic infections (Charalampous et al., 2019) and detection of pathogenic variants (Goenka et al., 2022).

However, due to the designed principle and data characteristics of NST (Magi et al., 2017), bioinformatics methods and tools developed based on NST have not been entirely satisfactory in terms of their adaptability, accuracy, and robustness. Analytical tools for next-generation sequencing (NGS) cannot be directly used to analyze NST data, so there is an urgent need for specially designed and efficient NST bioinformatics analysis methods and toolkits to provide solutions for scientific research and clinical practice. This Research Topic includes four papers that explain the application of NST and related bioinformatics analysis tools from different perspectives, such as the Android App (KARGAMobile) for real-time portable analysis of antibiotic resistance genes (ARGs) Barquero et al., evaluation of *KIF20A* as a prognostic biomarker and therapeutic target for lung adenocarcinoma (LUAD) Sun et al., the detection method (NanoSTR) of target short tandem repeats (STRs) Lang et al. and improving the base-calling accuracy with data augmentation (Chen et al.).

Barquero et al. developed a mobile Android app, named KARGAMobile, which can be used for portable, real-time, and easy-to-interpret analysis of antibiotic resistance genes sequenced by NST. KARGAMobile uses a compressed ARG reference database and different internal data structures to save RAM usage, and has a user-friendly graphical interface that guides file browsing, loading, parameterization, and process execution. The output files are post-processed to create visual, printable, and shareable reports that help users interpret the results.

Sun et al. used TCGA and GTEx data to identify*KIF20A* as a hub gene for lung adenocarcinoma with comprehensive bioinformatics analysis. They found a negative correlation between *KIF20A* expression and overall survival, progression-free survival, and disease-free survival. They also found that *KIF20A* knockdown inhibited cell proliferation, induced G2/M phase arrest, and promoted apoptosis. Moreover, they speculated that *KIF20A*may be used as a prognostic biomarker and therapeutic target for LUAD.

Lang et al. developed NanoSTR, a method for target short tandem repeats detection based on NST. NanoSTR uses statistical methods such as “multi-sampling” and Length-Number-Rank (LNR) information with sequencing data to detect the target STR locus and genotyping. Compared with existing STR detection tools, NanoSTR showed higher accuracy, better performance, and certain robustness. NanoSTR not only avoided the problem of genotyping errors or inability caused by the NST data characteristics, but also eliminated the requirement of a genome background database construction or reference genome alignment, which could reduce the consumption of computing resources, and did not require secondary processing.

Chen et al. developed several data augmentation strategies for NST to reduce the size requirements of the dataset while improving the robustness, accuracy, and other performance of the basecalling algorithm. These data augmentation strategies could improve the accuracy of basecalling by more than 1% without doubling the size of the training dataset. This work not only reduced the time and computational costs consumed during development but also provided new insights into the new basecalling algorithm.

In conclusion, although there are not many papers in our Research Topic, we believe that these papers can provide help and reference to similar research fields, and we hope to promote the application of NST in more scientific research and practical scenarios.
